# Impact of Integrated Psychiatric and Psychotherapeutic Treatment on Perceived Anxiety and Severity of Depressive Symptoms

**DOI:** 10.3390/jcm14072175

**Published:** 2025-03-22

**Authors:** Jacek Maślankowski, Aleksandra Wolska, Katarzyna Bliźniewska-Kowalska, Andrzej Silczuk, Mariusz Gujski, Agata Szulc, Justyna Kunikowska, Małgorzata Gałecka

**Affiliations:** 1 Antoni Jurasz University Hospital No. 1, 85-094 Bydgoszcz, Poland; yacek.maslankowski@gmail.com; 2 Department of Social Psychology, Faculty of Psychology, Kazimierz Wielki University in Bydgoszcz, 85-867 Bydgoszcz, Poland; aleksandrawolska82@gmail.com; 3 Department of Adult Psychiatry, Medical University of Lodz, 92-213 Lodz, Poland; katarzyna.blizniewska-kowalska@umed.lodz.pl; 4 Department of Community Psychiatry, Faculty of Health Sciences, Warsaw Medical University, 02-091 Warsaw, Poland; andrzej.silczuk@wum.edu.pl; 5 Department of Public Health, Faculty of Health Sciences, Warsaw Medical University, 02-091 Warsaw, Poland; mariusz.gujski@wum.edu.pl; 6 Psychiatric Clinic, Faculty of Health Sciences, Warsaw Medical University, 02-091 Warsaw, Poland; agata.szulc@wum.edu.pl; 7 Specialized Psychiatric Health Care Center, J. Babiński Hospital, 91-229 Lodz, Poland; kunikowska.justyna@gmail.com; 8 Department of Psychotherapy, Medical University of Lodz, 91-229 Lodz, Poland

**Keywords:** psychotherapy, anxiety, depression

## Abstract

**Background/Objectives**: This study aimed to assess the impact of integrated psychiatric-psychotherapeutic treatment on anxiety levels, mental health perception, and depression severity. **Methods:** Patients with a diagnosis of depressive or anxiety disorders were included in the study. The group was randomly divided into two subgroups. The first was the study group (N = 32), which underwent a process of standardized integrated treatment, while the second was the control group (N = 32), whose course of treatment lacked a standardized integrated treatment involving direct cooperation between a psychiatrist and a psychotherapist. Both groups of patients participated in the study for a period of three months (12 weeks). All study participants (N = 64) had the following psychological questionnaires administered at three time points (at study enrollment, after 6 weeks of psychotherapy, and after 12 weeks): STAI, GHQ-28, and BDI-II. **Results**: The analysis showed a statistically significant beneficial effect of integrated psychiatric and psychotherapeutic treatment on anxiety (both state and trait), severity of depressive symptoms, and all dimensions measured on the GHQ-28 scale except somatic symptoms. In addition, comparing the impact of the integrated approach and standard psychotherapeutic methods, the analysis indicated a group × time interaction effect for the variable ‘trait anxiety’ (STAI) and ‘anxiety, insomnia’ (GHQ-28). **Conclusions**: The use of an integrated psychotherapeutic model can be beneficial in patients with anxiety and depressive symptoms.

## 1. Introduction

Integrated treatment in psychiatry and psychology is gaining importance in the context of a comprehensive approach to mental health. The integration of psychotherapy, pharmacotherapy, and psychosocial interventions enhances treatment outcomes. Studies show that a combination of these methods may be more effective in treating psychiatric disorders such as depression, anxiety, and personality disorders [1,2].

Integrated treatment implies a holistic approach to the patient that takes into account not only mental symptoms, but also psychosocial, biological, and environmental factors. This approach allows us to better understand the complexity of mental disorders and to tailor therapy to the individual needs of the patient [3]. Combining different treatment methods can also improve patients’ adherence to treatment recommendations, which is key to recovery [4].

Psychotherapy—including Cognitive Behavioral Therapy (CBT), Dialectical Behavioral Therapy (DBT), and psychodynamic therapies—plays an important role in integrated treatment. Studies indicate that psychotherapy can be as effective as pharmacotherapy in treating many mental disorders, and in some cases even surpass the effects of drug treatment [2,5]. In addition, psychotherapy can help patients develop coping skills and improve their quality of life.

Pharmacotherapy is often an essential part of the treatment of mental disorders, especially in cases of more severe depressive episodes or anxiety disorders. However, it is important that it is used in an integrated manner with psychotherapy, which can increase the effectiveness of treatment. Studies show that patients who receive both psychotherapy and medication have better outcomes than those who are treated with only one of these methods [6].

In addition to psychotherapy and pharmacotherapy, psychosocial interventions—such as group support, family therapy, and rehabilitation programs—can be an important part of integrated treatment. This approach focuses on strengthening the patient’s social support and improving their interpersonal skills [7]. These interventions can also help the patient cope with problems related to the patient’s daily life and reduce the stigma of people with mental disorders.

The aim of the study was to evaluate the impact of integrated psychiatric and psychotherapeutic treatment on the experience of anxiety, assessment of mental health dimensions, and severity of depressive symptoms, as well as to compare the impact of the integrated approach and standard psychotherapeutic methods on the aforementioned clinical symptoms.

## 2. Materials and Methods

### 2.1. Study Participants

Patients were qualified after a medical examination by a psychiatric specialist. The examination included patients with a diagnosis of depressive disorders (F32, F33 according to ICD-10) or anxiety (F40–F48 according to ICD-10) [8]. This group was randomly (through non-returnable random sampling) divided into two subgroups. The first subgroup was the study group, which underwent a process of standardized integrated treatment, while the second was the control group, whose course of treatment lacked standardized integrated treatment involving direct cooperation between a psychiatrist and a psychotherapist. Both groups of patients participated in the study for a period of three months.

#### 2.1.1. Inclusion Criteria

- Diagnosis of anxiety or depressive disorders, disease entity from F32, F33, and F40 to F48 according to the ICD 10 classification;
- Age range from 18 years old (patients before the age of 18 require a different specificity of therapeutic interventions that take into account the developmental process) up to the age of 60 (above 60 years of age, there are significant factors of a biological nature as well as those resulting from the stage of life that may interfere with the clinical picture of the study).

#### 2.1.2. Criteria for Exclusion from Participation in the Study

- Addiction to psychoactive drugs, abstinence lasting less than a year, no participation in addiction therapy during abstinence;
- The presence of psychotic symptoms;
- Organic disorders (caused by brain dysfunction, disease, damage, or injury);
- Long history of psychotherapeutic treatment (more than 3 years) with no visible progress; no motivation to take psychotherapeutic treatment.

The patients were under the constant care of a psychiatric specialist. They were undergoing drug treatment. The therapeutic or research process did not modify the psychiatric treatment.

### 2.2. Specifics of Psychotherapeutic Interventions

Psychotherapeutic treatment followed the psychodynamic paradigm; each session lasted 40 min and was held twice a week. The main premise of the paradigm is that the therapeutic relationship reflects the patient’s past experiences—the experiences that influence dysfunctional adaptive strategies. Therapeutic interventions expand the patient’s psychological knowledge and enable insights into the nature of past functioning and pro-health solutions to be made.

The standardization of integrated treatment established a reproducible structure based on a protocol that includes standardized time frames for assessment, a fixed cyclical schedule of patient-specialist meetings, a defined content framework for these meetings, and specific research instruments to be applied at various stages of the process.

### 2.3. Study Protocol

**Figure 1:**
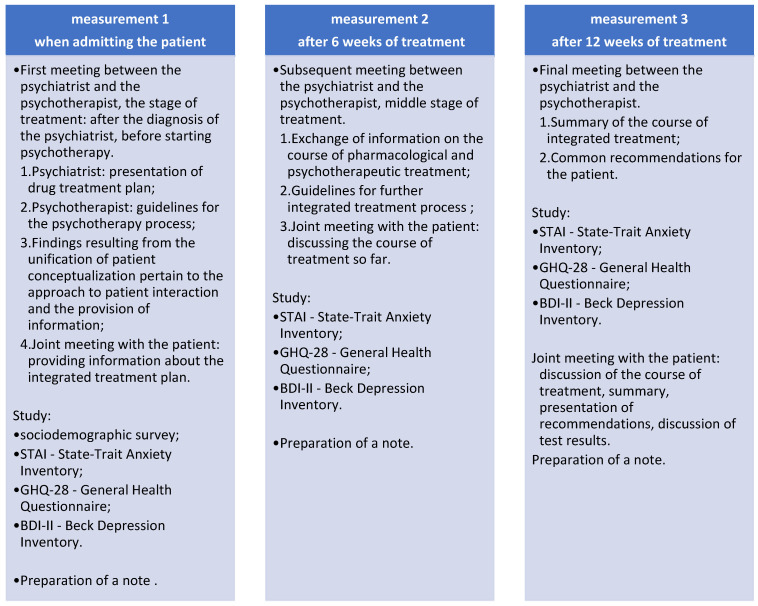
Study protocol.

The study protocol is shown in Figure 1.

### 2.4. Psychological Tool

On the day of inclusion in the study, all participants were asked to fill out a sociodemographic questionnaire, which included questions regarding among others age, education level, but also previous experiences and the reason for seeking psychotherapy.

In addition, all study participants had the following psychological questionnaires administered at three time points (at study inclusion, after 6 weeks of psychotherapy, and after 12 weeks):

STAI—The State-Trait Anxiety Inventory is a tool designed to measure anxiety understood as a transient and situationally conditioned state of an individual and anxiety understood as a relatively permanent personality trait. The STAI consists of two subscales, one of which (X1) is used to measure state anxiety and the other (X2) to measure trait anxiety. The questions that make up the two scales are placed on both sides of a single test sheet. Each subscale consists of 20 items, to which the respondent replies by selecting one of four categorized responses. Participants are asked to complete the inventory on day 0, in the middle part of the treatment/study, and on the day of the end of the study.

GHQ-28—The General Health Questionnaire is used to assess the mental health of adults. It provides a total score, which is an indicator of the patient’s mental health; however, the GHQ-28 version has four scales in addition to the total score: ‘somatic symptoms’, ‘anxiety, insomnia’, ‘social dysfunction’, and ‘severe depression’ to identify those whose mental state has broken down temporarily or long-term as a result of experienced difficulties, problems, or as a consequence of mental illness, and those at significant risk of mental health disorders.

BDI-II—The Beck Depression Inventory—Second Edition (BDI-II) is a self-report instrument consisting of 21 items. BDI-II is a tool for measuring the severity of depression in psychiatrically diagnosed patients. BDI-II was developed to indicate the presence and severity of depressive symptoms.

### 2.5. Statistical Analysis

To verify study hypotheses, the following statistical tests were used: Repeated Measures ANOVA Test, Bonferroni Post Hoc Test, and Pearson’s Chi-Square Test. Statistical analyses were conducted using the Statistica 13.3 software package. The first step involved verifying the assumption of normality in the distribution of quantitative variables for the entire sample. For this purpose, the Shapiro–Wilk test [9] was conducted. Variables for which the distribution analysis yielded statistical significance (*p* < 0.05) were considered to deviate from a normal distribution. The groups compared to each other in terms of the variable ‘gender’: n female = 21; n male = 11 (*chi*^2^ = 3.13; *p* = 0.077) were equal, while in terms of the variable ‘education’ they were not equal (*chi*^2^ = 22.50; *p* < 0.001) [10]. The variable ‘age’, as a quantitative variable, was examined for normality of distribution (results are presented in Table 1—the first table containing descriptive statistics). Additionally, other assumptions for statistical tests were verified, including homogeneity and sphericity of variance. Based on these verifications, appropriate statistical tests were selected to match the type of variables used in the hypotheses. At the beginning of the chapter, descriptive statistics for the dependent variables are presented, categorized by measurement time points.

## 3. Results

### 3.1. Characteristics of the Study Group

The study group, to which the integrated psychotherapeutic model was applied, consisted of 32 individuals aged 19 to 57 years (*M* = 32.38; *SD* = 11.51)—21 (65.5%) women and 11 (34.4%) men. Twenty-four (75.0%) of the subjects were single, one (3.1%) was divorced, seven (21.9%) were married. Nineteen (59.4%) of the subjects had secondary education, seven (21.9%) had vocational education, five (15.6%) had higher education, and one (3.1%) had primary education. A total of 15 participants (46.9%) reported the severity of experienced symptoms as the primary reason for seeking psychotherapy. Ten (31.3%) received a referral from a psychiatrist, while five (15.6%) cited insufficient previous pharmacological-only treatment as the reason. In contrast, one subject (3.1%) cited family expectations, and one (3.1%) mentioned curiosity about psychotherapy as their reason for seeking treatment. A total of 27 participants (84.4%) reported previous experience with psychotherapy, whereas five subjects (15.6%) had not previously undergone this form of treatment. The average duration of prior treatment was 16 months (*SD* = 19.91), with a minimum of 0 months and a maximum of 84 months (equivalent to 7 years). At the time of enrollment in the study, 31 subjects (96.9%) were undergoing pharmacotherapy, while only one (3.1%) was not taking any medication. The average duration of medication use in the study group was 32 months (*SD* = 38.19), with a minimum of 0 months and a maximum of 132 months (equivalent to 11 years). The characteristics of demographic variables, including gender, age, marital status, education, reason for seeking psychotherapy, experience with psychotherapy, duration of previous psychotherapy, medication use, and duration of medication use, are presented in the table below (Table 2).

### 3.2. Characteristics of the Control Group

The control group in the study consisted of 32 participants aged 19 to 58 years (*M* = 30.50; *SD* = 11.21)—26 (81.3%) women and six (18.7%) men. Twenty-five (78.1%) subjects were single, one (3.1%) was divorced, five (15.6%) were married, one (3.1%) was widowed. Seventeen (53.1%) participants had secondary education, one (3.1%) had vocational education, eight (25.0%) had higher education, and one (3.1%) had primary education. A total of 13 subjects (40.6%) reported the severity of experienced symptoms as the primary reason for seeking psychotherapy. Fourteen (43.8%) received a referral from a psychiatrist, while three (9.4%) cited insufficient previous pharmacological-only treatment as the reason. In contrast, two subjects (6.3%) cited family expectations as the reason for seeking psychotherapy. Previous experience with psychotherapy was declared by 28 respondents (87.5%), while four (12.5%) had not previously received this form of treatment. The average duration of previous treatment was 8.63 months (*SD* = 8.59), with a minimum of 0 months and a maximum of 36 months. Thirty-two (100.0%) subjects were on pharmacotherapy at the time of enrollment in the study. The average duration of medication use in the control group was 14.53 months (*SD* = 1.03), with a minimum of 0 months and a maximum of 40 months. The characteristics of demographic variables, including gender, age, marital status, education, reason for seeking psychotherapy, experience with psychotherapy, duration of previous psychotherapy, medication use, and duration of medication use, are presented in the tables below (Table 3).

The study group and the control group were comparable in terms of gender and age and did not differ significantly in a statistically meaningful way regarding the variables shown below (Table 4 and Table 5).

### 3.3. Assessment of the Effect of Integrated Psychiatric and Psychotherapeutic Treatment on Anxiety Perception and the Severity of Depressive Symptoms

Main research question: Does integrated treatment influence psychological variables, including state/trait anxiety, depression, somatic symptoms, insomnia, social dysfunction, and overall mental health? The tables below (Table 6, Table 7 and Table 8) present descriptive statistics for quantitative variables in the study group.

The Shapiro–Wilk test results for the analyzed quantitative variables across different time points indicated that some variables had distributions approximating normality (*p* > 0.005), while others deviated from normality (*p* < 0.005). Therefore, the skewness and kurtosis values of the analyzed variables were also examined. As a result, it was determined that these values exceeded the absolute value of 1 for only a few variables. The above distribution analyses justify the use of parametric tests for statistical analyses involving the variables presented in Tables.

The aggregated results of the statistical analysis are presented in the table below (Table 9). The dependent variables in the analyzed model were psychological variables, while the independent variable was measurement time.

#### 3.3.1. STATE ANXIETY (STAI)

**Figure 2:**
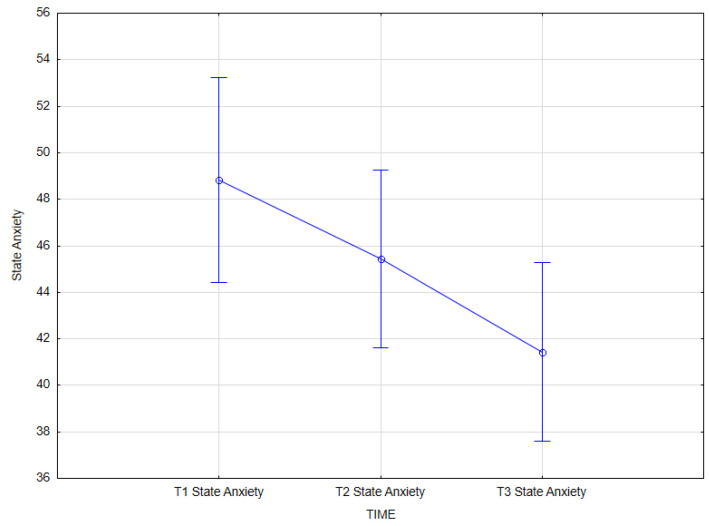
State anxiety at T1, T2, and T3 (times of measurement).

The analysis demonstrated the effect of integrated psychiatric and psychotherapeutic treatment on state anxiety (main effect *F*(2; 62) = 8.31; *p* = 0.001; *η^2partial^* = 0.211). The results of the Bonferroni test indicated that the improvement effect was statistically significant between the first measurement (*M* = 48.84; *SD* = 12.23) and the third measurement (*M* = 41.44; *SD* = 10.66) (*p* = 0.001) (Table 10; Figure 2).

#### 3.3.2. TRAIT ANXIETY (STAI)

The results of the statistical analysis showed that there is an effect of integrated psychiatric and psychotherapeutic treatment on trait anxiety (main effect *F*(2, 62) = 17.73; *p* < 0.001; *η^2partial^* = 0.364).

**Figure 3:**
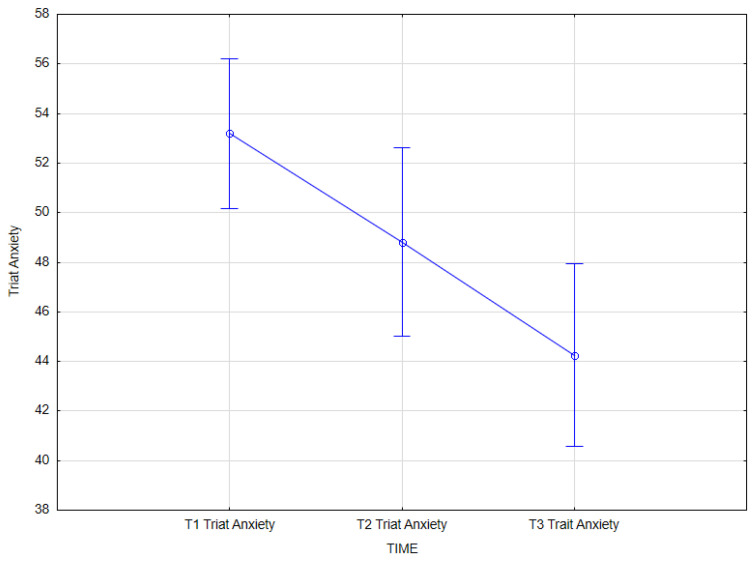
Trait anxiety at T1, T2, and T3 (times of measurement).

The results of the Bonferroni test indicated that the improvement effect was statistically significant between the first measurement (*M* = 53.19, *SD* = 8.40) and the second measurement (*M* = 48.81, *SD* = 10.53); *p* = 0.015, between the first measurement (*M* = 53.19, *SD* = 8.40) and the third measurement (*M* = 44.25, *SD* = 10.20); *p* < 0.001, and between the second measurement (*M* = 48.81, *SD* = 10.53) and the third measurement (*M* = 44.25, *SD* = 10.20); *p* = 0.010 (Table 11, Figure 3).

#### 3.3.3. DEPRESSION SEVERITY (BDI-II)

The analysis also demonstrated the effect of integrated psychiatric and psychotherapeutic treatment on the severity of depression as measured by BDI-II (main effect *F*(2, 62) = 17.00; *p* < 0.001; *η^2partial^* = 0.354).

**Figure 4:**
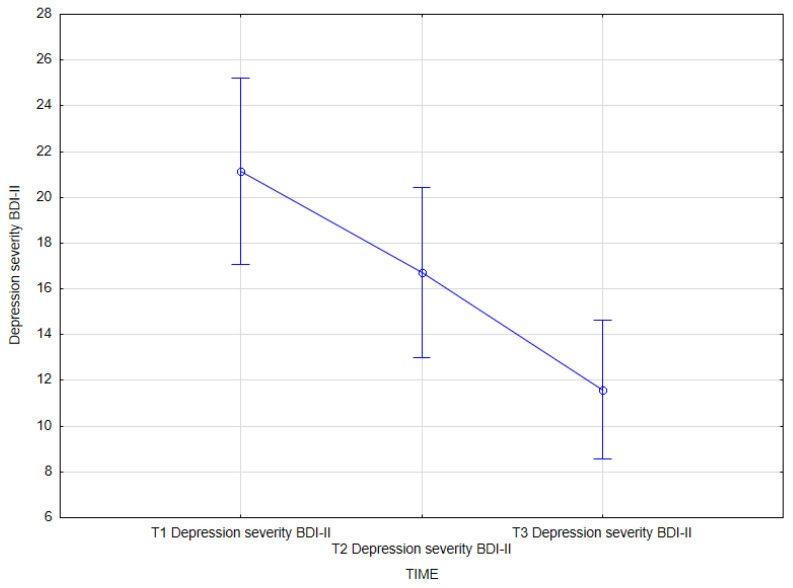
Depression severity (BDI-II) at T1, T2, and T3 (times of measurement).

The results of the Bonferroni test indicated that the improvement effect was statistically significant between the first measurement (*M* = 21.16; *SD* = 11.31) and the second measurement (*M* = 16.72; *SD* = 10.28); *p* = 0.027; between the first measurement (*M* = 21.16; *SD* = 11.31) and the third measurement (*M* = 11.59; *SD* = 8.40); *p <* 0.001; and between the second measurement (*M* = 16.72; *SD* = 10.28) and the third measurement (*M* = 11.59; *SD* = 8.40); *p =* 0.008 (Table 12, Figure 4).

#### 3.3.4. SOMATIC SYMPTOMS (GHQ-28)

The statistical analysis revealed no effect of integrated psychiatric and psychotherapeutic treatment on the level of the variable somatic symptoms (GHQ-28).

#### 3.3.5. ANXIETY, INSOMNIA (GHQ-28)

**Figure 5:**
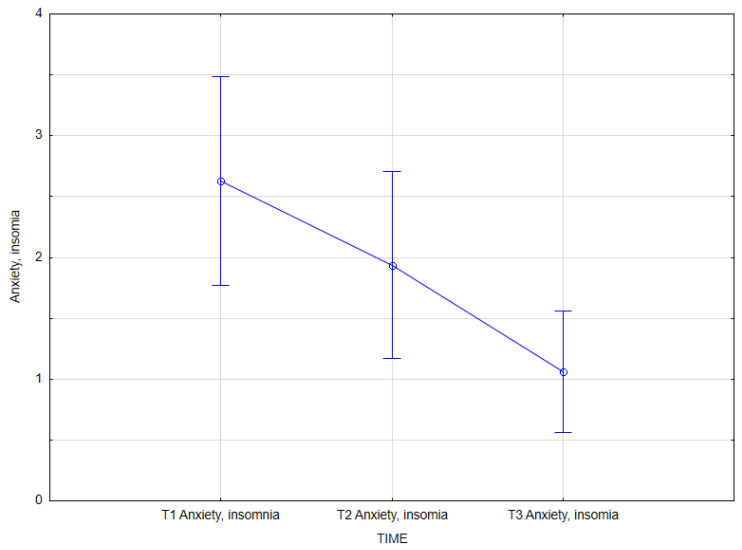
Anxiety, Insomnia at T1, T2, and T3 (times of measurement).

However, the effect of integrated psychiatric and psychotherapeutic treatment on anxiety, insomnia (GHQ-28) was revealed (main effect *F*(2, 62) = 8.73; *p* <0.001; *η^2partial^* = 0.219). The results of the Bonferroni test indicated that the improvement effect was statistically significant between the first measurement (*M* = 2.63, *SD* = 2.38) and the third measurement (*M* = 1.06, *SD* = 1.39); *p* < 0.001. The remaining differences were not statistically significant (Table 13, Figure 5).

#### 3.3.6. SOCIAL DYSFUNCTION (GHQ-28)

The statistical analysis revealed an effect of integrated psychiatric and psychotherapeutic treatment on social dysfunction (GHQ-28) (main effect *F*(2, 62) = 7.16; *p* = 0.002; *η^2partial^* = 0.187).

**Figure 6:**
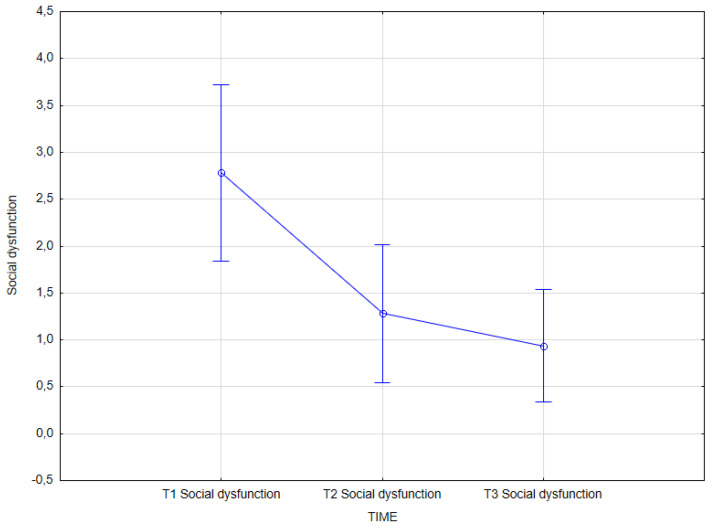
Social dysfunction at T1, T2, and T3 (times of measurement).

The results of the Bonferroni test indicated that the improvement effect was statistically significant between the first measurement (*M* = 2.78, *SD* = 2.60) and the second measurement (*M* = 1.28, *SD* = 2.04); *p* = 0.016, as well as between the first measurement (*M* = 2.78, *SD* = 2.60) and the third measurement (*M* = 0.94, *SD* = 1.66); *p* = 0.002. The remaining differences were not statistically significant (Table 14, Figure 6).

#### 3.3.7. SEVERE DEPRESSION (GHQ-28)

The statistical analysis revealed an effect of integrated psychological and psychiatric treatment on severe depression (GHQ-28) (main effect *F*(2, 62) = 10.09; *p* = 0.001, *η^2partial^* = 0.246).

**Figure 7:**
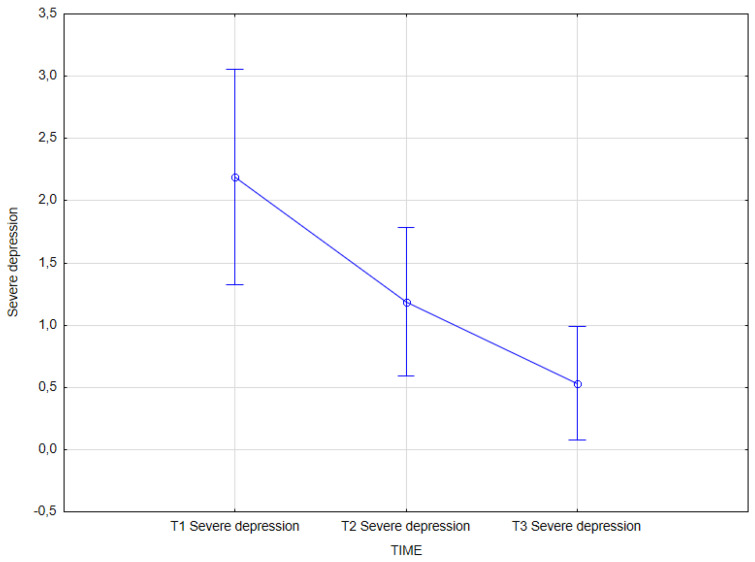
Severe depression at T1, T2, and T3 (times of measurement).

The results of the Bonferroni test indicated that the improvement effect was statistically significant between the first measurement (*M* = 2.19, *SD* = 2.40) and the second measurement (*M* = 1.19, *SD* = 1.65); *p* = 0.027, as well as between the first measurement (*M* = 2.19, *SD* = 2.40) and the third measurement (*M* = 0.53, *SD* = 1.27); *p* < 0.001. The remaining differences were not statistically significant (Table 15, Figure 7).

#### 3.3.8. MENTAL HEALTH ASSESSMENT (GHQ-28)

The final analysis revealed an effect of integrated treatment on mental health assessment (GHQ-28) (main effect *F*(2, 62) = 10.24; *p* = 0.001, *η^2partial^* = 0.248).

**Figure 8:**
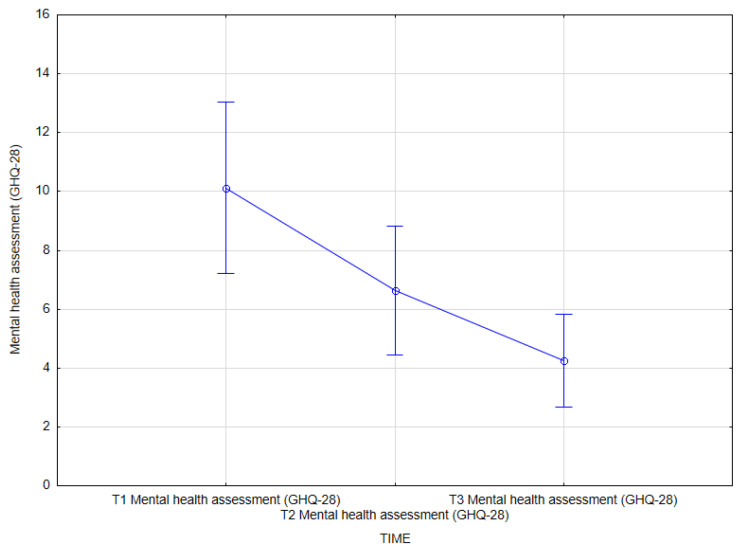
Mental health assessment (GHQ-28) at T1, T2, and T3 (times of measurement).

The results of the Bonferroni test indicated that the improvement effect was statistically significant between the first measurement (*M* = 10.13, *SD* = 8.10) and the second measurement (*M* = 6.63, *SD* = 6.07); *p* = 0.027, as well as between the first measurement (*M* = 10.13, *SD* = 8.10) and the third measurement (*M* = 4.25, *SD* = 4.41); *p* < 0.001. The remaining differences were not statistically significant (Table 16, Figure 8).

#### 3.3.9. Comparison of the Effects of Integrated Approaches and Standard Psychotherapeutic Methods on the Clinical Symptoms Analyzed

**Figure 9:**
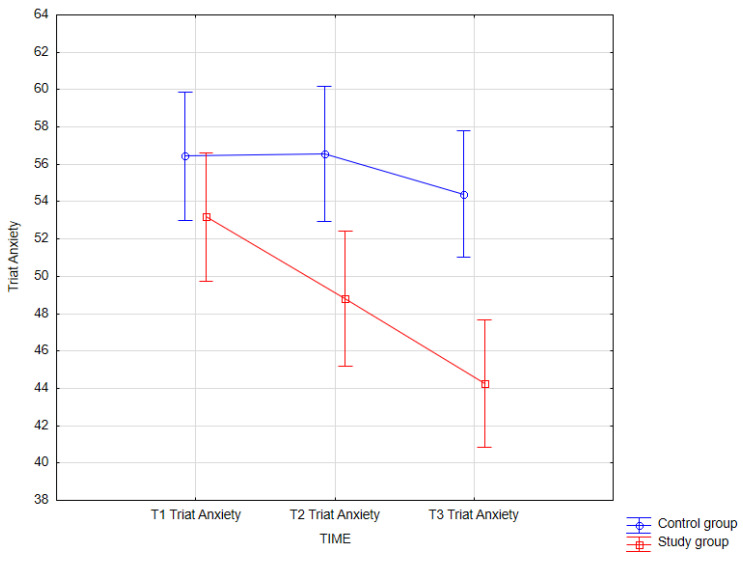
Trait anxiety—mixed in control and study groups at T1, T2, and T3 (times of measurement).

**Figure 10:**
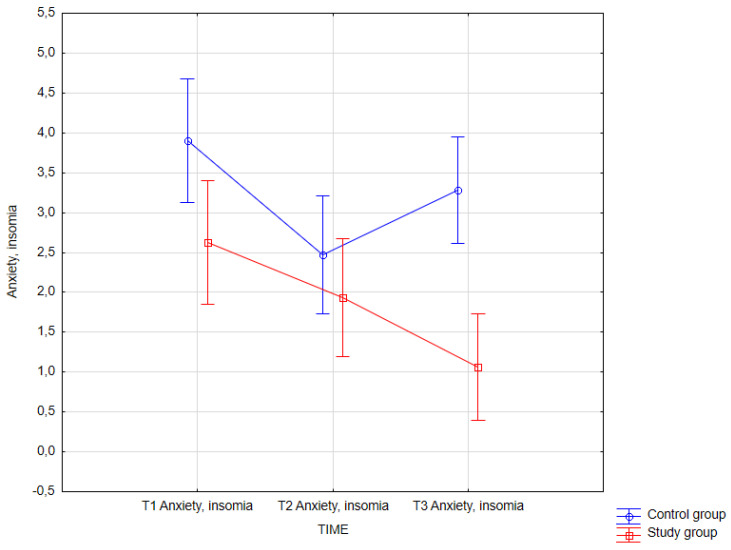
Anxiety, insomnia—mixed in control and study groups at T1, T2, and T3 (times of measurement).

The following tables (Table 17, Table 18, Table 19 and Table 20, Figure 9 and Figure 10) present the results of statistical analysis for both the control and study groups, considering measurement time. In the presented model, the dependent variables were psychological variables, and the independent variable was measurement time. The results shown in the tables below pertain to the dependent variables: trait anxiety (STAI X2) and anxiety, insomnia (GHQ-28).

The statistical analysis revealed an interaction effect between the factors ‘group’ and ‘time’ exclusively for these variables. This indicates that group membership significantly influenced the temporal changes observed in these psychological variables.

#### 3.3.10. Trait Anxiety

The main effect of the group factor was revealed as was the interaction effect of the two factors *F* (2, 124) = 4.14; *p* = 0.018. To conduct a detailed analysis of simple effects, a post hoc analysis was performed. The obtained results of the statistical analysis for the interaction of the factors are presented below.

The results of the Bonferroni test indicated that trait anxiety significantly differed (*p* < 0.001) between the third measurement in the study group (*M* = 44.25; *SD* = 10.20) and the first measurement in the control group (*M* = 56.44; *SD* = 10.91); between the third measurement in the study group (*M* = 44.25; *SD* = 10.20) and the second measurement in the control group (*M* = 56.56; *SD* = 9.93) (*p* < 0.001); between the third measurement in the study group (*M* = 44.25; *SD* = 10.20) and the third measurement in the control group (*M* = 54.41; *SD* = 8.97); and between the third measurement in the study group (*M* = 44.25; *SD* = 10.20) and the first measurement in the study group (*M* = 53.19; *SD* = 8.40).

Additionally, the second measurement of trait anxiety in the control group significantly differed (*p* = 0.037) from the first measurement in the control group (*M* = 56.44; *SD* = 10.91) and significantly differed (*p* = 0.031) from the second measurement in the control group (*M* = 56.56; *SD* = 9.93).

#### 3.3.11. Anxiety, Insomnia (GHQ-28)

The statistical analysis of the collected data revealed differences in the level of the variable anxiety, insomnia based on measurement time (main effect), group membership (control or study group) (main effect), and the interaction of both factors, *F*(2, 124) = 4.28; *p* = 0.016. To conduct a detailed analysis of simple effects, post hoc analyses were performed. The obtained results of the statistical analysis are presented below.

The results of the statistical analysis of simple effects indicated the presence of significant differences in the mean score of the variable ‘anxiety, insomnia’ between the second measurement in the control group (*M* = 2.47; *SD* = 2.06) and the third measurement in the study group (*M* = 1.06; *SD* = 1.39). The obtained result shows a statistically significant decrease (*p* < 0.001). The score on the second measurement of the control group significantly (*p* = 0.009) decreases relative to the first score in the control group (*M* = 3.91; *SD* = 1.99). The mean score of the variable ‘anxiety, insomnia’ in the control group increases at the third time measurement (*M* = 3.28; *SD* = 2.30) and is significantly higher than the third time measurement in the study group (*M* = 1.06; *SD* = 1.39). The last statistically significant result was revealed between the third time measurement in the study group (*p* = 0.003) and the first time measurement in the study group (*M* = 2.63; *SD* = 2.38).

## 4. Discussion

The integrated approach to psychiatric and psychological treatment offers numerous benefits, including improved therapeutic outcomes and greater patient satisfaction. Combining psychotherapy, pharmacotherapy, and psychosocial interventions can lead to holistic treatment of mental disorders. However, to fully realize the potential of this approach, it is necessary to overcome challenges related to its implementation and to promote collaboration among various specialists.

The process of integrated treatment has the following course. The first stage of cooperation between the psychiatrist and psychotherapist is contained in mutual recognition of the methods of cognition and curiosity about the difference in language. In turn, the mutual relationship requires the rejection of rivalry in favor of an attitude of cooperative partnership. The next stage is diagnosis, which is supposed to lead to the formulation of conceptualizations in nosological terms. In the integrated model, it is not only supported by psychological testing and observation, but is formed through a dialogue between psychiatric methodology and psychotherapeutic understanding. Professionals learn to tolerate some disorder and ambiguity while requiring a concretized nosological procedure. The result is an integrated indication for pharmacotherapy and an appropriate form of psychotherapy.

As is well known, psychotherapeutic treatment is carried out in diverse paradigms that derive from different philosophies of psychological understanding of human developmental stages. This, in turn, results in specific techniques. But even where the predominance of structuring tools with didactic threads is noticeable, the relationship becomes a basic module of the therapy process and an important healing factor. Thus, also in the triad psychiatrist–patient–psychotherapist, the relationship between these objects becomes a unifying factor, and its course and the patient’s participation with his experience of this triad acquires additional dimensions in gaining his understanding. For the treatment stage, standardization of integrated treatment becomes a necessary postulate, which should form a reproducible structure regardless of the dissimilarity of patients’ diagnoses. Different levels of anxiety and thus different intensity of defense mechanisms, from primitive to more mature levels, result in different patient perceptions of the same elements. The study of this dissimilarity is expected to create a system of recommendations for applying appropriate therapeutic interventions in the treatment process, increasing its effectiveness. The anticipated benefit is the isolation of significant factors for psychiatric-psychotherapeutic treatment that improves patients’ condition or reduces the duration of treatment. In addition, it creates conditions for communicative commonality in integrated treatment, increases the patient’s awareness of active self-understanding in medical and psychological aspects. Also, it reduces the risk in patients of perpetuating symptomatic language and persisting in secondary gains due to personality or anxiety disorders.

In such a model, which assumes the cooperation of a psychiatrist with a psychotherapist, patients with different needs will find their place, but also the combination of pharmacotherapy and psychotherapy will acquire a new dimension, and even the dissimilarity of therapists’ modalities will become functional. The team of specialists, despite the different tasks and competencies and complex projective processes in the relationship with patients, through the commonality of language improves the container functions, does not stop at the superficial level of conflict, and uncovers the hidden processes in the triad.

In the studies conducted by Cuijpers et al. (2016), it was found that the combination of Cognitive Behavioral Therapy (CBT) with pharmacotherapy significantly improves treatment outcomes in patients with depression. The authors observed that the integrated model leads to a more rapid reduction in depressive symptoms and long-term therapeutic effects [11]. The integrated approach also demonstrated effectiveness in the treatment of anxiety disorders. In the study by Hofmann et al. (2012), it was demonstrated that combined therapy—integrating Cognitive Behavioral Therapy (CBT) and pharmacotherapy—leads to a significant reduction in anxiety symptoms compared to pharmacotherapy alone [5].

Research suggests that the integrated model may also impact patients’ life satisfaction. In studies conducted by Seligman et al. (2005), it was found that therapies combining various methods contribute to an improvement in well-being and life satisfaction in patients with depression and anxiety [12,13].

Our study confirmed the effect of integrated psychiatric and psychotherapeutic treatment on anxiety levels, as measured by the STAI scale (main effect: *F*(2, 62) = 8.31; *p* = 0.001; *η^2partial^* = 0.211). This indicates an improvement in patients’ condition regarding anxiety levels, considered as a current emotional state. The effect of integrated psychiatric and psychotherapeutic treatment on trait anxiety was also demonstrated (main effect: *F*(2, 62) = 17.73; *p* < 0.001; *η^2partial^* = 0.364) [14], understood as a disposition of how to react. Thus far, little attention has been devoted by researchers to the analysis of changes in the intensity of state anxiety and trait anxiety during the course of psychotherapy. The limited number of publications available suggests that individual psychotherapy is effective in reducing the intensity of trait anxiety.

Numerous studies confirm the effectiveness of the integrated model in the treatment of depression. For example, a study published in *The Journal of Clinical Psychiatry* demonstrated that patients who received integrated treatment exhibited significantly lower levels of depressive symptoms compared to those who were treated with pharmacotherapy alone [15]. Similar findings were obtained in studies conducted by Johnson et al. (2022) [16], who observed that patients undergoing Cognitive Behavioral Therapy (CBT) in combination with antidepressant medication achieved better outcomes on the Hamilton Depression Rating Scale (HDRS).

Our analysis also demonstrated the effect of integrated psychiatric and psychotherapeutic treatment on the severity of depression, as measured by BDI-II (main effect: *F*(2, 62) = 17.00; *p* < 0.001; *η^2partial^* = 0.354). Similar results were obtained in the statistical analysis, which revealed an effect of integrated psychological and psychiatric treatment on severe depression measured by GHQ-28 (main effect: *F*(2, 62) = 10.09; *p* = 0.001. *η^2partial^* = 0.246).

The integrated model can operate at different levels. Pharmacotherapy can rapidly alleviate depressive symptoms, enabling the patient to be more receptive to psychotherapy. Psychotherapy, including Cognitive Behavioral Therapy (CBT), helps identify and modify negative thought patterns, which may lead to long-term improvement. Psychosocial interventions, such as group support or family therapy, can further enhance treatment outcomes, increasing the patient’s engagement in the recovery process (Garcia et al., 2023) [17]. The integrated psychiatric and psychological model demonstrates significant effectiveness in the treatment of depressive disorders. Collaboration among various specialists and a holistic approach to patient care contribute to better therapeutic outcomes and improved quality of life for patients. In light of the growing body of evidence supporting the effectiveness of this model, it is essential to further investigate and implement integrated treatment strategies in clinical practice.

Our findings demonstrate a significant effect of integrated treatment on anxiety and insomnia (GHQ-28) (*F*(2, 62) = 8.73; *p* < 0.001; *η^2partial^* = 0.219).

Insomnia is a frequently co-occurring issue in anxiety disorders. Numerous studies have demonstrated that psychotherapy can effectively reduce insomnia symptoms. In research conducted by Morin, LeBlanc, Daley, Gregoire, and Mérette (2006) [18], it was found that Cognitive Behavioral Therapy for Insomnia (CBT-I) provides significant benefits in improving sleep quality. The authors observed that psychotherapeutic interventions led to a reduction in sleep onset latency, an increase in total sleep time, and an improvement in subjective sleep quality. Additionally, a study conducted by van Straten, Frijters, Bergström, Huibers, Koole, and Andersson (2018) [19] confirmed that Cognitive Behavioral Therapy for Insomnia (CBT-I) is effective in treating chronic insomnia, with its effects persisting even after the completion of therapy.

At the final stage of the analysis, the effect of integrated treatment on mental health assessment (GHQ-28 total score) was revealed (main effect: *F*(2, 62) = 10.24; *p* = 0.001, *η^2partial^* = 0.248). This finding is consistent with the earlier study conducted by Kobys et al. (2020), which demonstrated that patients receiving integrated treatment experience significant improvements in mental health compared to those receiving traditional care. The study included patients with depression and anxiety disorders, and the results indicated a reduction in depressive and anxiety symptoms [20].

Our study, which conducted a comparative analysis between the study group, treated using the integrated psychiatric and psychological model, and the control group, which followed a traditional psychotherapeutic model without collaboration with a psychiatrist or pharmacotherapy, revealed statistically significant differences in trait anxiety as measured by the STAI questionnaire, as well as in experienced anxiety and insomnia. These findings confirm the significant impact of close interdisciplinary collaboration and an integrated conceptualization of the patient on key aspects of mental health functioning.

Despite the advantages of the integrated approach, there are numerous challenges associated with its implementation. Successful application requires cooperation among various specialists, which can be difficult to achieve in practice [13]. Furthermore, differences in approach and professional philosophy between psychiatrists and psychotherapists may lead to conflicts, which could have a negative impact on patients. Therefore, it is crucial for therapeutic teams to be well-organized and to maintain effective communication to ensure continuity of care.

As this was an observational study, in which participation in the study could not affect the pharmacotherapy used, it should be taken into account that the type of biological treatment used may have been some kind of bias factor.

The lack of subgroup analysis constitutes the limitations of our study. It would be worth considering performing such a deeper analysis in follow-up studies on a larger number of patients.

## 5. Conclusions

Both psychological aspects such as the concept of affective temperaments [21] and biological aspects play an important role in the development of mood and anxiety disorders, hence the importance of an integrated approach in the treatment of these disorders as well [22].

The integrated psychiatric and psychotherapeutic model shows promising results in the treatment of depressive and anxiety disorders. Further research in this area is warranted to gain a deeper understanding of the mechanisms underlying this approach and its long-term therapeutic effects.

The integration of various therapeutic methods within a single model may be key to achieving better clinical outcomes and improving patients’ quality of life [22].

## Figures and Tables

**Table 1:** Education.

Education	Number	Percentage
b	19	59.4
c	7	21.9
a	5	15.6
d	1	3.1

Basic statistics for the variable ‘education’ (*n* = 32), *chi*^2^ = 22.50; *p* < 0.001. Source: own elaboration.

**Table 2:** Basic statistics for the variables ‘gender‘, ‘marital status’, ‘education’, ‘reason for seeking psychotherapy’, ‘experience with psychotherapy’, ‘medication use’, ‘age’, ‘duration of previous psychotherapy (months)’, ‘and duration of medication use (months)’. Study group (n = 32).

Gender	Number		%		Basic Statistics for the Variable			
					*chi* ^2^ *(df)*			*p* Value
Female	21		65.5		3.13 (1)			0.077
Male	11		34.4					
Marital status	Number		%		*chi* ^2^ *(df)*			*p* Value
Single	24		75.0		26.69 (2)			<0.001
Divorced	1		3.1					
Married	7		21.9					
Education	Number		%		*chi* ^2^ *(df)*			*p* Value
Secondary	19		59.4		22.50 (3)			<0.001
Vocational	7		21.9					
Higher	5		15.6					
Primary	1		3.1					
Reason for seeking psychotherapy	Number		%		*chi* ^2^ *(df)*			*p* Value
Increase in symptom severity	15		46.9		23.00 (4)			<0.001
Referral from a psychiatrist	10		31.3					
Inadequate previous pharmacological-only treatment	5		15.6					
Family expectations	1		3.1					
Interest in psychotherapy	1		3.1					
Experience with psychotherapy	Number		%		*chi* ^2^ *(df)*			*p* Value
Participation in psychotherapy for less than one year	8		25.0		4.75 (3)			0.191
Participation in only a few sessions so far	6		18.8					
Participation in psychotherapy for more than one year	13		40.6					
No experience	5		15.6					
Medication use	Number		%		*chi* ^2^ *(df)*			*p* Value
Yes	31		96.9		28.13 (1)			<0.001
No	1		3.1					
	*M*	*Min*	*Max*	*SD*	*As*	*K*	*W*	*p* value
Age	32.38	19.00	57.00	11.51	0.77	−0.66	0.89	0.003
Duration of previous psychotherapy (months)	16.09	0.00	84.00	19.91	1.80	3.48	0.78	<0.001
Duration of medication use (months)	32.25	0.00	132.00	38.19	1.75	1.93	0.72	<0.001

Source: own elaboration. Explanations: %—percentage; *M*—mean; *SD*—standard deviation; *As*—skewness; *K*—kurtosis; *Min*—minimum value; *Max*—maximum value; *W*—Shapiro–Wilk test result; *p*—statistical significance.

**Table 3:** Basic statistics for the variables ‘gender’, ‘marital status’, ‘education’, ‘reason for seeking psychotherapy’, ‘experience with psychotherapy’, ‘medication use’, ‘age’, ‘duration of previous psychotherapy (months)’, and ‘duration of medication use (months)’. Control group (n = 32).

Gender		Number		%		Basic Statistics for the Variable		
						*chi* ^2^ *(df)*		*p* Value
Female		26		81.3		37.5 (1)		<0.001
Male		6		18.7				
Marital status		Number		%		*chi* ^2^ *(df)*		*p* Value
Single		25		78.6		49.50 (3)		<0.001
Divorced		1		3.1				
Married		5		15.6				
Widowed		1		3.1				
Education		Number		%		*chi* ^2^ *(df)*		*p* Value
Secondary		17		53.1		16.75 (3)		0.001
Vocational		6		18.8				
Higher		8		25.0				
Primary		1		3.1				
Reason for seeking psychotherapy		Number		%		*chi* ^2^ *(df)*		*p* Value
Increase in symptom severity		13		40.6		15.25 (3)		0.002
Referral from a psychiatrist		14		43.8				
Inadequate previous pharmacological-only treatment		3		9.4				
Family expectations		2		6.3				
Experience with psychotherapy		Number		%		*chi* ^2^ *(df)*		*p* Value
Participation in psychotherapy for less than one year		6		18.8		45.75 (3)		0.124
Participation in only a few sessions so far		9		28.1				
Participation in psychotherapy for more than one year		13		40.6				
No experience		4		12.5				
Medication use		Number		%		*chi* ^2^ *(df)*		*p* Value
Yes		32		100.0		-		-
	*M*	*Min*	*Max*	*SD*	*As*	*K*	*W*	*p* value
Age	30.50	19.00	58.00	11.21	0.79	−0.54	0.87	0.001
Duration of previous psychotherapy (months)	8.63	0.00	36.00	8.59	1.33	1.98	0.86	0.001
Duration of medication use (months)	14.53	0.00	40.00	1.03	1.75	0.82	0.90	0.006

Source: own elaboration. Explanations: %—percentage; *M*—mean; *SD*—standard deviation; *As*—skewness; *K*—kurtosis; *Min*—minimum value; *Max*—maximum value; *W*—Shapiro–Wilk test result; *p*—statistical significance.

**Table 4:** Comparison of the control and study groups in terms of the variables ‘gender’ and ‘age’.

Study Group	Control Group 0—Male	Control Group 1—Female	Total
0—male	2	9	11
1—female	4	17	21
Total	6	26	32

Comparison of control and study group size by gender (*n* = 32), *chi*^2^ = 0.004; *df* =1; *p* =0.952 *(p* = 0.670 Fisher’s exact test). Source: own elaboration.

**Table 5:** Basic descriptive statistics for the variable ‘age’.

Variable	*M*	*Min*	*Max*	*SD*	*As*	*K*	*W*	*p*
Age, control group	30.50	19.00	58.00	11.21	0.79	−0.54	0.87	0.001
Age, study group	32.38	19.00	57.00	11.51	0.77	−0.66	0.89	0.003

Source: own elaboration. Explanations: *M*—mean; *SD*—standard deviation; *As*—skewness; *K*—kurtosis; *Min*—minimum value; *Max*—maximum value; *W*—Shapiro–Wilk test result; *p*—statistical significance. The value *p* for comparison of means, two-sided test = 0.511.

**Table 6:** Basic descriptive statistics for the variables with the result of the Shapiro–Wilk test (*n* = 32), first measurement.

Variable	*M*	*Min*	*Max*	*SD*	*As*	*K*	*W*	*p*
State anxiety	48.84	27.00	74.00	12.23	0.19	−0.58	0.98	0.670
Trait anxiety	53.19	34.00	71.00	8.40	0.31	0.42	0.96	0.272
Depression total, BDI-II	21.16	0.00	56.00	11.31	0.69	1.62	0.96	0.314
Somatic symptoms	2.53	0.00	7.00	1.88	0.58	−0.17	0.93	0.052
Anxiety, insomnia	2.63	0.00	7.00	2.38	0.45	−1.14	0.89	0.003
Social dysfunction	2.78	0.00	7.00	2.60	0.41	−1.33	0.85	<0.001
Severe depression	2.19	0.00	7.00	2.40	0.84	−0.54	0.83	<0.001
Mental health assessment total, GHQ-28	10.13	0.00	27.00	8.10	0.56	−0.91	0.92	0.020
Age	32.38	19.00	57.00	11.51	0.77	−0.66	0.89	0.003

Source: own elaboration. Explanations: *M*—mean; *SD*—standard deviation; *As*—skewness; *K*—kurtosis; *Min*—minimum value; *Max*—maximum value; *W*—Shapiro–Wilk test result; *p*—statistical significance.

**Table 7:** Basic descriptive statistics for the variables with the result of the Shapiro–Wilk test (*n* = 32), second measurement.

Variable	*M*	*Min*	*Max*	*SD*	*As*	*K*	*W*	*p*
State anxiety	45.44	27.00	66.00	10.56	0.13	−0.66	0.96	0.340
Trait anxiety	48.81	29.00	77.00	10.53	0.61	0.51	0.97	0.559
Depression total, BDI-II	16.72	1.00	37.00	10.28	0.32	−0.94	0.96	0.236
Somatic symptoms	2.22	0.00	6.00	1.77	0.35	−1.09	0.90	0.007
Anxiety, insomnia	1.94	0.00	7.00	2.12	0.88	−0.28	0.84	<0.001
Social dysfunction	1.28	0.00	7.00	2.04	1.92	2.62	0.65	<0.001
Severe depression	1.19	0.00	5.00	1.65	1.23	0.17	0.74	<0.001
Mental health assessment total, GHQ-28	6.63	0.00	20.00	6.07	0.89	−0.47	0.87	0.001

Source: own elaboration. Explanations: *M*—mean; *SD*—standard deviation; *As*—skewness; *K*—kurtosis; *Min*—minimum value; *Max*—maximum value; *W*—Shapiro–Wilk test result; *p*—statistical significance.

**Table 8:** Basic descriptive statistics for the variables with the result of the Shapiro–Wilk test (*n* = 32), third measurement.

Variable	*M*	*Min*	*Max*	*SD*	*As*	*K*	*W*	*p*
State anxiety	41.44	27.00	67.00	10.66	0.81	−0.10	0.93	0.030
Trait anxiety	44.25	28.00	72.00	10.20	0.77	0.28	0.95	0.104
Depression total, BDI-II	11.59	1.00	33.00	8.40	1.34	1.12	0.85	<0.001
Somatic symptoms	1.72	0.00	6.00	1.76	1.06	0.48	0.85	<0.001
Anxiety, insomnia	1.06	0.00	4.00	1.39	1.04	−0.27	0.76	<0.001
Social dysfunction	0.94	0.00	6.00	1.66	2.03	3.77	0.63	<0.001
Severe depression	0.53	0.00	6.00	1.27	3.10	10.97	0.49	<0.001
Mental health assessment total, GHQ-28	4.25	0.00	19.00	4.41	1.40	2.49	0.85	0.001

Source: own elaboration. Explanations: *M*—mean; *SD*—standard deviation; *As*—skewness; *K*—kurtosis; *Min*—minimum value; *Max*—maximum value; *W*—Shapiro–Wilk test result; *p*—statistical significance.

**Table 9:** Effects of integrated psychiatric and psychotherapeutic treatment on psychological variables.

Dependent Variable	T1 (*n* = 32)		T2 (*n* = 32)		T3 (*n* = 32)		*F*	*df*	*P*	*η^2partial^*
	*M*	*SD*	*M*	*SD*	*M*	*SD*				
State anxiety	48.84	12.23	45.44	10.56	41.44	10.66	8.31	2, 62	0.001	0.211
Trait anxiety	53.19	8.40	48.81	10.53	44.25	10.20	17.73	2, 62	<0.001	0.364
Depression total, BDI-II	21.16	11.31	16.72	10.28	11.59	8.40	17.00	2, 62	<0.001	0.354
Somatic symptoms	2.53	1.88	2.22	1.77	1.72	1.76	2.14	2, 62	0.127	0.065
Anxiety, insomnia	2.63	2.38	1.94	2.12	1.06	1.39	8.73	2, 62	<0.001	0.219
Social dysfunction	2.78	2.60	1.28	2.04	0.94	1.66	7.16	2, 62	0.002	0.187
Severe depression	2.19	2.40	1.19	1.65	0.53	1.27	10.09	2, 62	0.001	0.246
Mental health assessment total, GHQ-28	10.13	8.10	6.63	6.07	4.25	4.41	10.24	2, 62	0.001	0.248

Source: own elaboration. Explanations: *F*—*F*-statistic value; *df*—degrees of freedom; *η^2partial^*—effect size; *p*—statistical significance.

**Table 10:** Bonferroni test results for comparisons between T1, T2, and T3 for the dependent variable ‘state anxiety’. Probabilities for post hoc tests.

Dependent Variable: State Anxiety	T1 (*n* = 32)		T2 (*n* = 32)		T3 (*n* = 32)	
	*M*	*SD*	*M*	*SD*	*M*	*SD*
	48.84	12.23	45.44	10.56	41.44	10.66
T1			0.197		0.001	
T2	0.197				0.095	
T3	0.001		0.095			

Source: own elaboration. Explanations: *M*—arithmetic mean; *SD*—standard deviation; T1, T2, T3—measurement time; *n*—sample size.

**Table 11:** Bonferroni test results for comparisons between T1, T2, and T3 for the dependent variable ‘trait anxiety’. Probabilities for post hoc tests.

Dependent Variable: Trait Anxiety	T1 (*n* = 32)		T2 (*n* = 32)		T3 (*n* = 32)	
	*M*	*SD*	*M*	*SD*	*M*	*SD*
	53.19	8.40	48.81	10.53	44.25	10.20
T1			0.015		<0.001	
T2	0.015				0.010	
T3	<0.001		0.010			

Source: own elaboration. Explanations: *M*—arithmetic mean; *SD*—standard deviation; T1, T2, T3—measurement time; *n*—sample size.

**Table 12:** Bonferroni test results for comparisons between T1, T2, and T3 for the dependent variable ‘depression severity (BDI-II)’. Probabilities for post hoc tests.

Dependent Variable: Depression Severity (BDI-II)	T1 (*n* = 32)		T2 (*n* = 32)		T3 (*n* = 32)	
	*M*	*SD*	*M*	*SD*	*M*	*SD*
	21.16	11.31	16.72	10.28	11.59	8.40
T1			0.027		<0.001	
T2	0.027				0.008	
T3	<0.001		0.008			

Source: own elaboration. Explanations*: M*—arithmetic mean; *SD*—standard deviation; T1, T2, T3—measurement time; *n*—sample size.

**Table 13:** Bonferroni test results for comparisons between T1, T2, and T3 for the dependent variable ‘anxiety, insomnia’. Probabilities for post hoc tests.

Dependent Variable: Anxiety, Insomnia	T1 (*n* = 32)		T2 (*n* = 32)		T3 (*n* = 32)	
	*M*	*SD*	*M*	*SD*	*M*	*SD*
	2.63	2.38	1.94	2.12	1.06	1.39
T1			0.214		<0.001	
T2	0.214				0.069	
T3	<0.001		0.069			

Source: own elaboration. Explanations: *M*—arithmetic mean; *SD*—standard deviation; T1, T2, T3—measurement time; *n*—sample size.

**Table 14:** Bonferroni test results for comparisons between T1, T2, and T3 for the dependent variable ‘social dysfunction’. Probabilities for post hoc tests.

Dependent Variable: Social Dysfunction	T1 (*n* = 32)		T2 (*n* = 32)		T3 (*n* = 32)	
	*M*	*SD*	*M*	*SD*	*M*	*SD*
	2.78	2.60	1.28	2.04	0.94	1.66
T1			0.016		0.002	
T2	0.016				1.000	
T3	0.002		1.000			

Source: own elaboration. Explanations: *M*—arithmetic mean; *SD*—standard deviation; T1, T2, T3—measurement time; *n*—sample size.

**Table 15:** Bonferroni test results for comparisons between T1, T2, and T3 for the dependent variable ‘severe depression’. Probabilities for post hoc tests.

Dependent Variable: Severe Depression	T1 (*n* = 32)		T2 (*n* = 32)		T3 (*n* = 32)	
	*M*	*SD*	*M*	*SD*	*M*	*SD*
	2.19	2.40	1.19	1.65	0.53	1.27
T1			0.027		<0.001	
T2	0.027				0.246	
T3	<0.001		0.246			

Source: own elaboration. Explanations: *M*—arithmetic mean; *SD*—standard deviation; T1, T2, T3—measurement time; *n*—sample size.

**Table 16:** Bonferroni test results for comparisons between T1, T2, and T3 for the dependent variable ‘mental health assessment (GHQ-28)’. Probabilities for post hoc tests.

Dependent Variable: Mental Health Assessment (GHQ-28)	T1 (*n* = 32)		T2 (*n* = 32)		T3 (*n* = 32)	
	*M*	*SD*	*M*	*SD*	*M*	*SD*
	10.13	8.10	6.63	6.07	4.25	4.41
T1			0.027		<0.001	
T2	0.027				0.246	
T3	<0.001		0.246			

Source: own elaboration. Explanations: *M*—arithmetic mean; *SD*—standard deviation; T1, T2, T3—measurement time; *n*—sample size.

**Table 17:** The impact of integrated psychiatric and psychotherapeutic treatment on the psychological variable of trait anxiety, as measured by the State-Trait Anxiety Inventory (STAI X2), repeated measures designs, effects, and power.

Trait Anxiety—STAI X2	*F*	*p*	η^2partial^	*df*
Group	12.13	<0.001	0.164	1
Measurement time	10.30	<0.001	0.145	2
Group × measurement time	4.14	0.018	0.063	2

Source: own elaboration. Explanations*: F*—*F*-statistic value; *df*—degrees of freedom; *η^2partial^*—effect size; *p*—statistical significance.

**Table 18:** Simple comparisons, factor: measurement time and group, dependent variable: trait anxiety (STAI X-2).

Trait Anxiety (STAI X2)			(1)		(2)		(3)		(4)		(5)		(6)	
			*M*	*SD*	*M*	*SD*	*M*	*SD*	*M*	*SD*	*M*	*SD*	*M*	*SD*
Subclass no.	gr	T	56.44	10.91	56.56	9.93	54.41	8.97	53.19	8.40	48.81	10.53	44.25	10.20
1	K	T1			1.000		1.000		1.000		0.037		<0.001	
2	K	T2	1.000				1.000		1.000		0.031		<0.001	
3	K	T3	1.000		1.000				1.000		0.376		0.001	
4	B	T1	1.000		1.000		1.000				0.185		<0.001	
5	B	T2	0.037		0.031		0.376		0.185				0.137	
6	B	T3	<0.001		<0.001		0.001		<0.001		0.137			

Source: own elaboration. Explanations: *M*—arithmetic mean; *SD*—standard deviation; T1, T2, T3—measurement time; *K*—control group, *B*—study group; *n*—sample size.

**Table 19:** Effect of integrated psychiatric and psychotherapeutic treatment on the psychological variable ‘anxiety, insomnia’, repeated measures designs, effects, and power.

Anxiety, Insomnia	*F*	*p*	*η^2partial^*	*df*
group	11.61	0.001	0.158	1
Measurement time	9.28	<0.001	0.130	2
Group × measurement time	4.28	0.016	0.065	2

Source: own elaboration. Explanations: *F*—*F*-statistic value; *df*—degrees of freedom; *η^2partial^*—effect size; *p*—statistical significance.

**Table 20:** Simple comparisons, factor: measurement time and group, dependent variable: anxiety, insomnia.

Anxiety, Insomnia			(1)		(2)		(3)		(4)		(5)		(6)	
			*M*	*SD*	*M*	*SD*	*M*	*SD*	*M*	*SD*	*M*	*SD*	*M*	*SD*
Subclass no.	gr	T	3.91	1.99	2.47	2.06	3.28	2.30	2.63	2.38	1.94	2.12	1.06	1.39
1	K	T1			0.009		1.000		0.214		0.003		<0.001	
2	K	T2	0.009				0.736		1.000		1.000		0.109	
3	K	T3	1.000		0.736				1.000		0.154		<0.001	
4	B	T1	0.214		1.000		1.000				1.000		0.003	
5	B	T2	0.003		1.000		0.154		1.000				0.514	
6	B	T3	<0.001		0.109		<0.001		0.003		0.514			

Source: own elaboration. Explanations: *M*—arithmetic mean; *SD*—standard deviation; T1, T2, T3—measurement time; *K*—control group, *B*—study group; *n*—sample size.

## Data Availability

The data presented in this study are available on request from the corresponding author due to privacy and ethical reasons.
